# The impact of national and international travel on spatio-temporal transmission of SARS-CoV-2 in Belgium in 2021

**DOI:** 10.1186/s12879-023-08368-9

**Published:** 2023-06-24

**Authors:** Minh Hanh Nguyen, Thi Huyen Trang Nguyen, Geert Molenberghs, Steven Abrams, Niel Hens, Christel Faes

**Affiliations:** 1 Data Science Institute, I-BioStat, Hasselt University, BE-3500 Hasselt, Belgium; 2 I-BioStat, Katholieke Universiteit Leuven, BE-3000 Leuven, Belgium; 3 Global Health Institute, University of Antwerp, BE-2000 Antwerpen, Belgium; 4 Centre for Health Economic Research and Modelling Infectious Diseases, Vaccine and Infectious Disease Institute, University of Antwerp, BE-2000 Antwerpen, Belgium

**Keywords:** Spatio-temporal model, COVID-19, Human mobility, International travel

## Abstract

**Background:**

The Severe Acute Respiratory Syndrome Coronavirus 2 (SARS-CoV-2) has rapidly spread over the world and caused tremendous impacts on global health. Understanding the mechanism responsible for the spread of this pathogen and the impact of specific factors, such as human mobility, will help authorities to tailor interventions for future SARS-CoV-2 waves or newly emerging airborne infections. In this study, we aim to analyze the spatio-temporal transmission of SARS-CoV-2 in Belgium at municipality level between January and December 2021 and explore the effect of different levels of human travel on disease incidence through the use of counterfactual scenarios.

**Methods:**

We applied the endemic-epidemic modelling framework, in which the disease incidence decomposes into endemic, autoregressive and neighbourhood components. The spatial dependencies among areas are adjusted based on actual connectivity through mobile network data. We also took into account other important factors such as international mobility, vaccination coverage, population size and the stringency of restriction measures.

**Results:**

The results demonstrate the aggravating effect of international travel on the incidence, and simulated counterfactual scenarios further stress the alleviating impact of a reduction in national and international travel on epidemic growth. It is also clear that local transmission contributed the most during 2021, and municipalities with a larger population tended to attract a higher number of cases from neighboring areas.

**Conclusions:**

Although transmission between municipalities was observed, local transmission was dominant. We highlight the positive association between the mobility data and the infection spread over time. Our study provides insight to assist health authorities in decision-making, particularly when the disease is airborne and therefore likely influenced by human movement.

**Supplementary Information:**

The online version contains supplementary material available at 10.1186/s12879-023-08368-9.

## Background

**Fig. 1:**
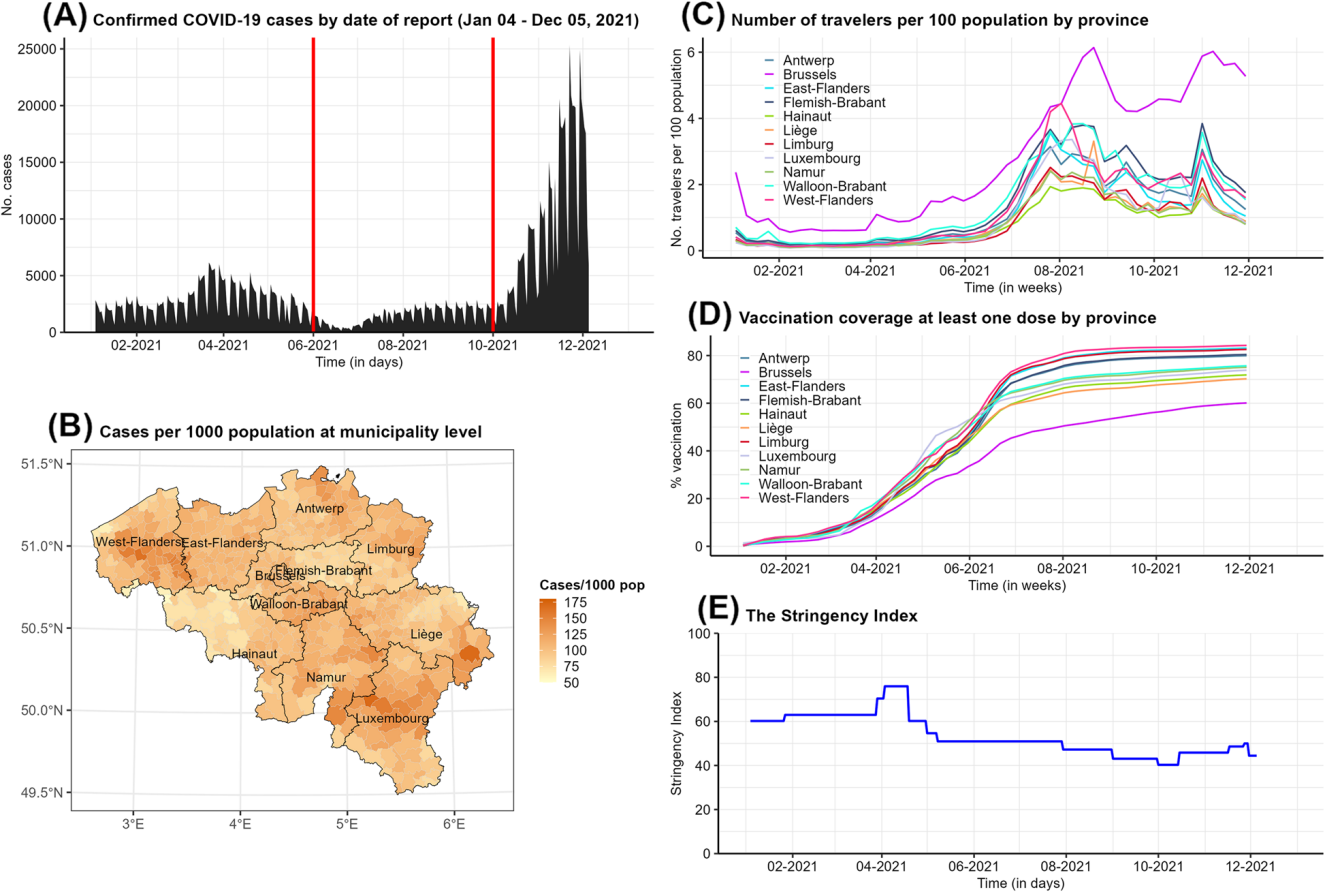
Distribution of reported cases by date (**A**) and municipality (**B**) and time-dependent data on international travelers per 100 inhabitants (**C**), vaccination coverage of at least one dose of COVID-19 vaccine (**D**), and stringency index (**E**) in Belgium from Week 2021-1 (04/1/2021) to Week 2021-48 (05/12/2021). Two vertical red lines in (**A**) are to distinguish between the three time periods considered in our analyses: January - May, June - September, and October - December 2021

The emergence and rapid worldwide spread of the Severe Acute Respiratory Syndrome Coronavirus 2 (SARS-CoV-2) and its implied Coronavirus Disease 2019 (COVID-19) has tremendously impacted global health. Several studies suggested that the transmission dynamics of SARS-CoV-2 are explained by several spatio-temporal factors, including demographic factors [1], contact networks of individuals [2], containment measures [3, 4], and human mobility [5, 6]. In Belgium, approximately 650,000 COVID-19-positive cases were confirmed in 2020, and this number increased to 1.5 million confirmed cases over the course of 2021, accounting for 12.9% of the Belgian population. Cases were heterogeneously distributed across municipalities (see, e.g., Fig. 1). Few studies have examined the effects of control strategies and mobility on the spatial spread of COVID-19 in the country, and these studies have focused on waves in 2020 [2, 7, 8]. A study incorporating spatio-temporal heterogeneous factors (e.g., demographic factors, containment measures, human mobility) is therefore advisable to understand the transmission dynamics of SARS-CoV-2 in Belgium, especially in 2021.

Human mobility has been identified earlier as a quintessential factor in the spread of airborne infections [9–12]. Hence, to mitigate SARS-CoV-2 spread in Belgium, one of the early responses was the installment of severe restrictions on travel, both at the national and international level. Specifically, international travel was banned during the first wave, March-May 2020, and non-essential foreign travel to and from Belgium was prohibited between January 27 and April 19, 2021. However, being a small, centrally located European country, the relatively large in-flux of travelers - a fraction of which hosting the infection - especially in periods with little or no travel restrictions (e.g., from September 2021), potentially has an important impact on viral spread. The human mobility data in Belgium is available at several space-time resolutions. In our study, two main sources are used: summary data on national mobility from the mobile network and the number of incoming travelers to the country. As these data reflect changes in mobility patterns at different time periods, they can be used to describe disease spread. More specifically, the key question arises whether we use the available travel data to quantify the impact of travel on the spread of COVID-19, in order to use them as an important factor to simulate and predict the disease incidence in different time period.

In response to the pandemic, the non-pharmaceutical interventions (NPIs) issued by the Belgian government have changed with time, leading to variations in the stringency of restriction measures. In 2021, relatively stringent measures were still in place, but these no longer included lockdown and full school closure, although there were still longer periods with mask mandates (e.g., in public transport) and closure of culture and event sectors. A precise estimation of their effects on the evolution of infections is challenging in the face of sparse data availability. Fortunately, the Oxford Coronavirus Government Tracker (OxCGRT) provides a proxy metric, the so-called stringency index, to assess the level of strictness of social policies imposed at a national scale [13]. The stringency index has shown to be useful in recent spatio-temporal studies [14, 15]. Furthermore, vaccination campaigns have largely contributed to an important reduction of the risk of infection and of more severe outcomes such as hospitalization and death [16–19]. During 2021, mass vaccination campaigns were widely implemented in the Belgian population. However, vaccination coverages were heterogeneous across Belgian geographical units (see, e.g., Fig. 1D, and Faes et al. (2022) [20]). For pathogens such as measles, earlier findings indicated that the heterogeneity in vaccination coverage was associated with disease incidence [21, 22]. Notwithstanding, the impact of vaccination and the relation between vaccine uptake in the population and disease incidence may be different between different vaccine-preventable diseases. Besides, given the importance of demographic characteristics, which are known to vary by location and usually available in spatial resolution, it could not be neglected in studying the transmission process of COVID-19. Therefore, we expect that the combination of space-time available data will help to better unravel the complex dynamics of SARS-CoV-2, in this paper with a focus on Belgium.

Spatio-temporal modelling of infectious disease spread based on routine incidence data has received great interest in recent years. In the context of COVID-19, various modeling frameworks have been employed. For example, compartmental models (e.g., SEIR, metapopulation models) study the interaction between individuals in communities at different spatial scales [2, 23]. Spatial and temporal point processes have also been used to study the disease’s dynamics. Additional information like demographics, human mobility, and control measures have been considered as possible factors influencing transmission [24–26]. However, these models can be complex, require high-resolution data that may not always be available (e.g., susceptible proportion in communities), and can be time-consuming to compute. An adequate method is offered by a multivariate time series model in a so-called endemic-epidemic (EE) statistical modelling framework introduced by Held et al. (2005) [27]. An important aspect is that it employs a common regression framework, and the model’s parameters can be conveniently estimated using standard optimization techniques, such as maximum likelihood. In essence, the time-space dependence of infections is determined by both background risk independent of the epidemic and auto-regression on past counts of within- and between-geographical units. The flexibility of the EE framework enables us to include potential variables and model their effects on disease dynamics, in both time and space. Properly tailored models have been used to investigate and predict the evolution of the COVID-19 pandemic  [3, 14, 15, 28].

In this paper, we examine both temporal and spatial patterns in disease spread and the way their dynamics are influenced by human mobility, the stringency of NPIs, and the effectiveness thereof. We apply the EE modelling framework with the aim to (i) analyze the spatio-temporal transmission of SARS-CoV-2 in Belgium between January and December 2021 and (ii) explore the effect of different levels of human travel on disease incidence through the use of counterfactual scenarios. We leverage the available data on the daily time series for case incidence, national mobility between Belgian municipalities, the number of incoming international travelers to the country, the stringency of containment policies, vaccination coverage, and demographic variables. This study aims to provide public health authorities with essential information to make informed policy decision for mitigating the impact of COVID-19 as well as that of future pandemics, especially in relation to travel restrictions.

## Methods

In this section, we first describe the data sources used in our analyses. A summary of the data used can be found in Table 1. Then, we introduce a spatial dynamic model to assess the impact of travel rate on COVID-19 spread. This impact is further illustrated through counterfactual scenarios with different travel levels. Lastly, sensitivity analyses to assess the stability of model estimates are described.

**Table 1:** Overview of the different datasets used in the analyses with details on the resolution, spatial level and time range

Dataset	Resolution	Spatial level	Time range
Case data	Daily	Municipality	Jan 04 - Dec 05, 2021
Local mobility data	Daily	Municipality	Sep 01, 2020 - Jun 6, 2021
Stringency Index	Daily	National	Jan 04 - Dec 05, 2021
International travel data	Weekly	Province	Week 1 - Week 48, 2021
Vaccination data	Weekly	Municipality	Week 1 - Week 48, 2021
Population data	Yearly	Municipality	2020

### Data

#### Case data

The daily number of confirmed COVID-19 cases in each of the 581 municipalities in Belgium was retrieved for the period between January 4, 2021 and December 5, 2021 from Sciensano, the Scientific Institute for Public Health in Belgium. Figure 1A shows the evolution of the Belgian COVID-19 epidemic during 2021. More specifically, the total number of reported cases over time shows two waves: (1) a wave between January and June, 2021, representing the third COVID-19 wave in Belgium, and (2) a wave starting from September 2021 onwards. The daily number of cases started to increase again from July onwards and fluctuated around 2,500 confirmed cases before dramatically soaring to approximately 25,000 cases per day at the beginning of December. Furthermore, the lower left panel (Fig. 1B) shows the distribution of confirmed cases by Belgian municipality using heat colors. For areas that are darker, the number of confirmed cases is higher.

#### National and international mobility data

**Fig. 2:**
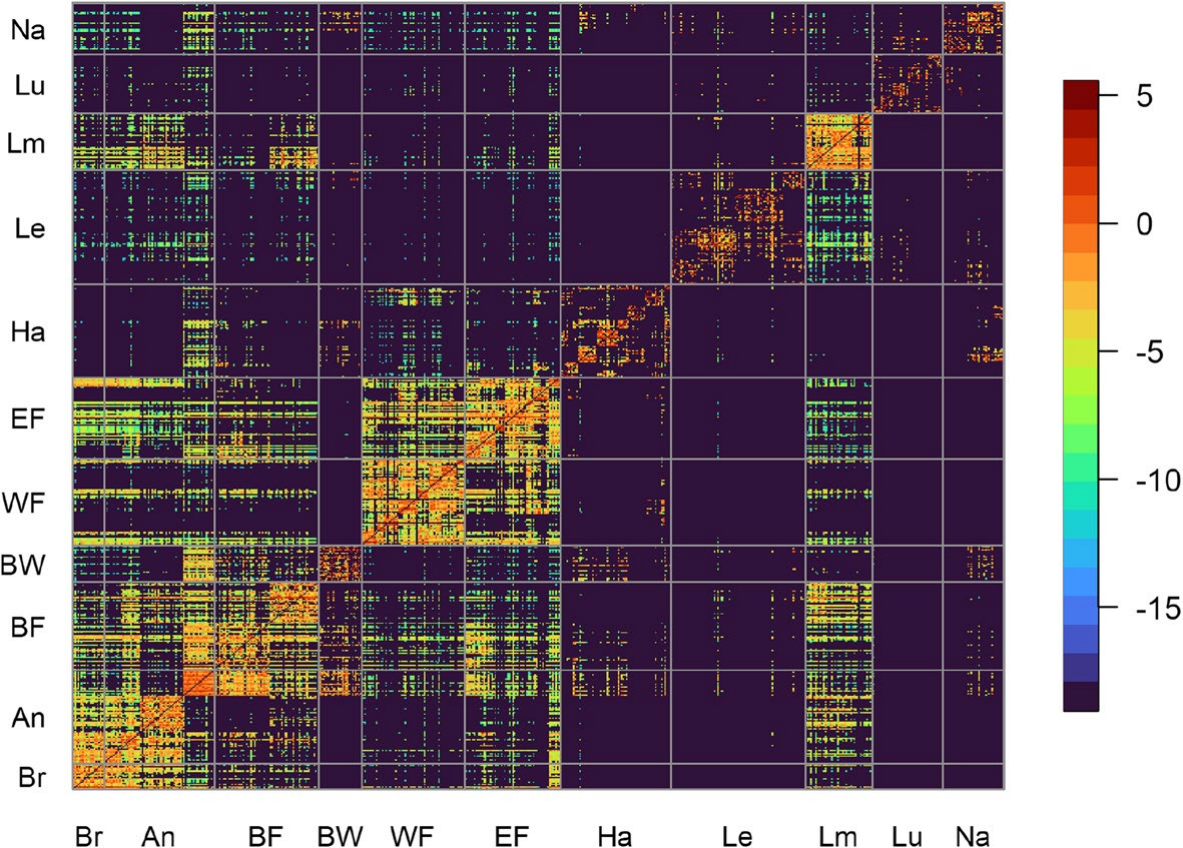
National mobility between September 1, 2020 and June 6, 2021. The *y*-axis represents the origin while the *x*-axis is the destination. Different municipalities are grouped according to the 10 Belgian provinces and Brussels-Capital Region (Brussels (Br), Antwerp (An), Flemish Brabant (BF), Walloon Brabant (BW), West Flanders (WF), East Flanders (EF), Hainaut (Ha), Liège (Le), Limburg (Lm), Luxembourg (Lu), and Namur (Na)

To assess the amount of travel between municipalities in Belgium, summary information from mobile network data is used, available between September 1, 2020 and June 6, 2021 [29]. These data provide information about the average proportion of time that customers spent in their residing municipality as well as in other municipalities (if any). Based on this, we can present the connectivity among municipalities through a mobility matrix (Fig. 2), calculated as the log-transformed mean percentage time spent in a municipality between September 2020 and June 2021, and averaged this temporal information to obtain a static network. The monthly mobility matrices over this time period are graphically depicted in Fig. A6 in the Appendix.

The international travel rate is available at the provincial level as the number of incoming travelers per 100 inhabitants. This information is based on the COVID-19 weekly reports by Sciensano. Figure 1C shows the time trend over the study period. The rate for all provinces was at the minimum during February—April 2021, when a travel ban on non-essential travel was in place (from January 27 until April 19, 2021). After this period, travel rates started to increase, to reach a peak around August and November 2021. Brussels remained the region with the highest travel rate during 2021.

#### Pharmaceutical and non-pharmaceutical interventions

The number of individuals that received at least one dose of a COVID-19 vaccine in 2021 was obtained from Sciensano [30]). Note that these data are available at municipality level and in weekly resolution. The proportion of vaccinated individuals in each Belgian province is depicted in Fig. 1D. Differences in vaccine coverage among provinces are clearly visible. West-Flanders had the highest proportion of individuals receiving at least one vaccine dose, while the lowest level was observed in Brussels.

The Stringency Index ranges from 0 to 100 and reflects the level of severity of non-pharmaceutical mitigation measures imposed at the national level (Oxford Coronavirus Government Tracker 2022) [13]. It is a composite measure of nine response metrics, including school and workplace closures, cancellation of public events, limitations on public gatherings, closure of or restrictions on public transport, stay-at-home mandates, public awareness campaigns, restrictions on internal mobility, and international movement control. Higher values of the Stringency Index imply that stricter measures were in place. Figure 1E presents the evolution of this index for Belgium throughout 2021. The highest values were observed during February–April 2021, with a peak in April 2021, at the time of the third wave.

#### Population data

The population data of each Belgian municipality in 2020 was retrieved from the Belgian statistical office [31]. The total population size was approximately 11.5 million individuals and was assumed to be constant during 2021.

### Spatial dynamic model

Let $Y_{it}$ denote the number of confirmed COVID-19 cases in municipality *i* ($i = 1, \ldots , 581$) on day *t* ($t = 1, \ldots , T$), with *T* the length of the time window considered in this analysis. Covariate information is represented by $\varvec{x}_{it}$. Assuming that the number of new confirmed cases $Y_{it}$ at day *t* depends on the (series of) past observations $Y_{i(t-d)}$, $d = 1, \ldots , D$, with up to $D = 7$ days being of importance to determine $Y_{it}$, the spatio-temporal model is formulated as [27, 32, 33]:

$$[Y_{it} \mid {Y}_{i(t-1)},\dots ,{Y}_{i(t-D)}, \varvec{x}_{it}] \sim \text {NegBin}(\mu _{it}, \psi ),$$

with mean incidence $\mu _{it}$ and variance $\mu _{it}(1+\psi \mu _{it})$. Here $\psi \ge 0$ is the dispersion parameter to be estimated. Note that if $\psi = 0$, mean and variance are equal and $Y_{it}$ follows a Poisson distribution. The conditional mean $\mu _{it}$ is modelled as:

1 $$\begin{aligned} \mu _{it} = \epsilon _{it} + {\lambda _{it}{\sum \limits _{d=1}^Du_{d}{Y_{i(t-d)}}}}+ {\phi _{it} \left( {\sum \limits _{d=1}^D\sum \limits _{j\ne i}u_{d}w_{{ji}}^*Y_{j(t-d)}}\right) }. \end{aligned}$$

The natural logarithm of the first term, referred to as the *endemic component* $\epsilon _{it}$, and interpreted as the background risk, equals

2 $$\begin{aligned} \log (\epsilon _{it}) = \log \left( N_i^*\right) + {\alpha }^{(\epsilon )} + {\beta }^{(\epsilon )}{\text{ WE }}_{t} + {\delta }^{(\epsilon )}{\text{ sus }}_{it} + {\eta _1}^{(\epsilon )}{\text{ travel }}_{it} + {\eta _2}^{(\epsilon )}{\text{ travel }}_{it}^2 + {\kappa }^{(\epsilon )}{\text{ SI }}_{t} +{\sum \limits _{k = 1}^{2}{ \rho }_{k}^{(\epsilon )}}{\varvec{1} }_{\{t \in \text {period }k\}}(t), \end{aligned}$$

with $N_i^* = {N_i}/{100,000}$ defining an offset. The second term is the *local epidemic* or *autoregressive component*, describing the local epidemic spread in time. The autoregressive parameter $\lambda _{it}$, is modeled as

3 $$\begin{aligned} \log (\lambda _{it}) = {\alpha }^{(\lambda )} + {\beta }^{(\lambda )}{\text{ WE }}_{t} + {\gamma }^{(\lambda )}{\log ({N}_{i})}+ {\delta }^{(\lambda )}{\text{ sus }}_{it} + {\eta }^{(\lambda )}{\text{ travel }}_{it} +{\eta _2}^{(\lambda )}{\text{ travel }}_{it}^2 + {\kappa }^{(\lambda )}{\text{ SI }}_{t} +{\sum \limits _{k = 1}^{2}{ \rho }_{k}^{(\lambda )}}{\varvec{1} }_{\{t\in \text {period }k\}}(t). \end{aligned}$$

Finally, the third term of mean $\mu _{it}$ represents the *global epidemic* or *neighbourhood component*. Its corresponding parameter $\phi _{it}$ is modeled as

4 $$\begin{aligned} \log (\phi _{it}) = {\alpha }^{(\phi )} + {\beta }^{(\phi )}{\text{ WE }}_{t} + {\gamma }^{(\phi )}{\log ({N}_{i})}+ {\eta }^{(\phi )}{\text{ travel }}_{it} +{\eta _2}^{(\phi )}{\text{ travel }}_{it}^2 + {\kappa }^{(\phi )}{\text{ SI }}_{t} +{\sum \limits _{{k = 1}}^{{2}}{ \rho }_{k}^{(\phi )}}{\varvec{1} }_{\{t\in \text { period }k\}}(t). \end{aligned}$$

Two types of weights are included in the model. First, the normalized Poisson weights $u_d$ in (1) represent the probability for a serial interval (i.e., the average time (in days) between symptom onset in an infectious individual and symptoms emerging in a newly infected individual when both were in close contact) of length *d* days with *d* taking values up to $D = 7$ days [33]. Second, the weights $w_{ji}^*$ in the neighbourhood component express the strength of connectivity between pairs of municipalities. The definition of the mobility weights is based on the estimated connectivity over time inferred from mobility data, and a comparison is made from defining the connectivity merely on the distance between two municipalities.

In the model, we include component-specific intercepts ($\alpha ^{(\epsilon )}$, $\alpha ^{(\lambda )}$, $\alpha ^{(\phi )}$). We account for various covariates, including a weekend effect (*WE*), population size (*N*) (in the local and global epidemic components), proportion of non-vaccinated individuals as a proxy for susceptibility (*sus*), travel rate (*travel*), stringency index (*SI*), and calendar time (*period*). Specifically, two time periods, i.e., from June to September 2021 and from October to December 2021, are considered in the analysis in order to distinguish between different phases in the epidemic. The effect of the proportion of susceptible individuals (*sus*) is accommodated in the model by considering the natural logarithm of the proportion of residents that are non-vaccinated (i.e., the fraction of individuals without any of the COVID-19 vaccine doses) [21, 22]. We add a quadratic term of the travel rate which was centered prior to inclusion in the model.

### Inference and model comparison

Model comparison is done based on the Akaike Information Criteria (AIC) [34]. The model with the lowest AIC is selected. Parameter inference is based on maximum likelihood estimation in R package surveillance [35] version 1.20.1 and hhh4addon version 0.0.0.0.9014 [33] under R version 4.0.5 [36].

### Counterfactual simulation of case counts

To investigate the impact of travel, we consider three scenarios with different levels of travel and simulate the expected number of cases. In the first scenario, the national mobility and international travel rate is the same as observed. In the second one, the international travel rate is set at the lowest level during the study period, while national mobility is kept the same as observed. In the third case, we show what the situation would be if there would be no mobility within the country.

### Sensitivity analyses

In order to check the stability of our model estimates, we performed four sensitivity analyses. First, the travel rate is estimated based on the number of incoming travellers from the Passenger Locator Form (PLF) data (Appendix Fig. A1). This form was required to be filled in by all passengers entering Belgium during the study period. Second, we use serial intervals based on a literature review. This includes a gamma distribution with shape and rate parameters equal to 2.29 and 0.36, respectively [37], and a lognormal distribution with parameters 1.38 and 0.563 [38]. Third, the Stringency Index was adjusted to exclude travel restrictions (i.e., restrictions on internal movement and international travel controls) (Appendix Fig. A2), in order to reduce dependencies between the (original) Stringency Index versus the national mobility and the international travel rate in our model. Finally, we represent municipality connectivity by a power law matrix instead of mobility matrices [39].

## Results

**Fig. 3:**
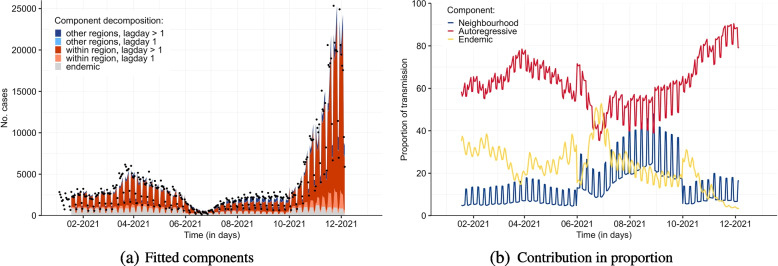
The left panel (**a**) shows the fitted components of the selected model on COVID-19 cases in Belgium; the dots indicate the observed number of daily confirmed cases. The right panel (**b**) depicts the proportions of transmission that can be attributed to each of the three components (i.e., endemic, autoregressive and neighbourhood)

Figure 3 depicts the model fit (panel (a)) and the contribution of each component (panel (b)). It can be seen that our model provides a good overall fit to the observed epidemic curve. Nevertheless, the estimated case numbers from around November onward are not as close to the observed data points as in the previous period. This may result as a result of the emergence of the Omicron variant, which started circulating in Belgium from December 2021 onward. The relative contribution of each component to the estimated daily number of cases is illustrated in the right panel of the same figure. The local epidemic spread component usually accounts for the largest part of the circulation, throughout the study period. A sudden increase in the share of the global spread was observed in June - September 2021. Overall, the endemic, local epidemic and global epidemic components accounted for 15.8%, 73.9%, and 10.3% of the estimated cumulative number of cases, respectively.

The exponentiated model parameters, representing the multiplicative effect at the original scale, with their 95% confidence intervals, are presented in Table 2. As expected, the number of confirmed cases in weekends is lower as compared to weekdays due to reporting delays, under-reporting, or delays in health-seeking behaviour. Municipalities with a larger population size have a stronger spatial epidemic spread while its effect on within-municipality epidemic spread is found to be insignificant. An increase in the proportion of susceptible individuals (as approximated by the fraction of non-vaccinated individuals) tends to lead to an increase in the number of confirmed cases, albeit that the effect is also insignificant. Interestingly, implementation of stricter measures is associated with a higher disease spread even though the effect is minimal. The two periods (i.e., June to September, 2021 and October to December, 2021) show different effects (compared to the reference period January to May, 2021) in each of the three model components. Both periods have a lower mean baseline incidence as compared to the first period (January to May, 2021). In the period of June to September, the speed of local spread is reduced as compared to in the reference period, while in the period October to December it increases again to a level which is higher than January to May, 2021. At the same time, the speed of disease spread between municipalities is elevated in the period of June to September as compared to the period of January to May, and not significantly different between the period of October to December and the period of January to May. Similar results are observed in the sensitivity analyses.

**Fig. 4:**
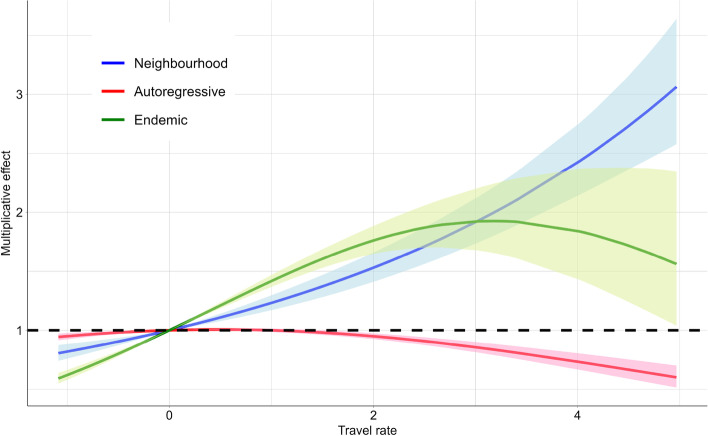
Impact of travel rate (proportion of incoming travelers per 100 population, centered) on the number of cases. The lines and envelopes are the mean estimate and 95%CI for the endemic (green), autoregressive (red) and neighbourhood component (blue)

The impact of travel rate on the mean number of cases is visualized in Fig. 4. Note that since the travel rate is centered, a value of zero on the *x*-axis corresponds to the mean travel rate on the original scale. From Fig. 4, it can be seen that the baseline circulation of COVID-19 gets higher if there is more international travel, and in addition that the spatial spread is higher when international travel increases. Indeed, in the endemic and neighbourhood components the baseline mean incidence (assuming a mean centered travel rate of zero) increases multiplicatively with a factor evolving in a non-linear way in relation to the travel rate, while the opposite (although albeit small) effect holds for the autoregressive component (red line). Furthermore, for large travel rates the increase in multiplicative effect with increasing travel rates stabilizes in the endemic case (green line), thereby deviating from an exponential increase in case of a linear travel rate term at the linear predictor scale.

**Table 2:** The exponentiated model parameter estimates with corresponding 95% confidence intervals (CIs)

	Local epidemic			Global epidemic			Endemic		
	Estimate	95% CI		Estimate	95% CI		Estimate	95% CI	
Intercept	0.78	0.68	0.89	0.03	0.02	0.05	4.73	3.67	6.09
Weekend	0.32	0.31	0.32	0.87	0.80	0.94	0.35	0.33	0.37
Population	0.99	0.98	1.00	1.14	1.10	1.19	-	-	-
Susceptible proportion	1.00	0.98	1.03	-	-	-	1.05	0.98	1.12
Travel rate	1.03	1.01	1.05	1.23	1.15	1.31	1.51	1.44	1.59
Travel rate$^2$	0.97	0.97	0.98	1.00	0.99	1.02	0.94	0.92	0.96
Stringency Index	1.00	1.00	1.01	1.01	1.01	1.02	1.01	1.01	1.02
Month Jun-Sep	0.88	0.85	0.92	2.01	1.70	2.36	0.29	0.27	0.31
Month Oct-Dec	1.45	1.39	1.52	1.09	0.91	1.32	0.80	0.71	0.89
AIC	754224.7								

**Fig. 5:**
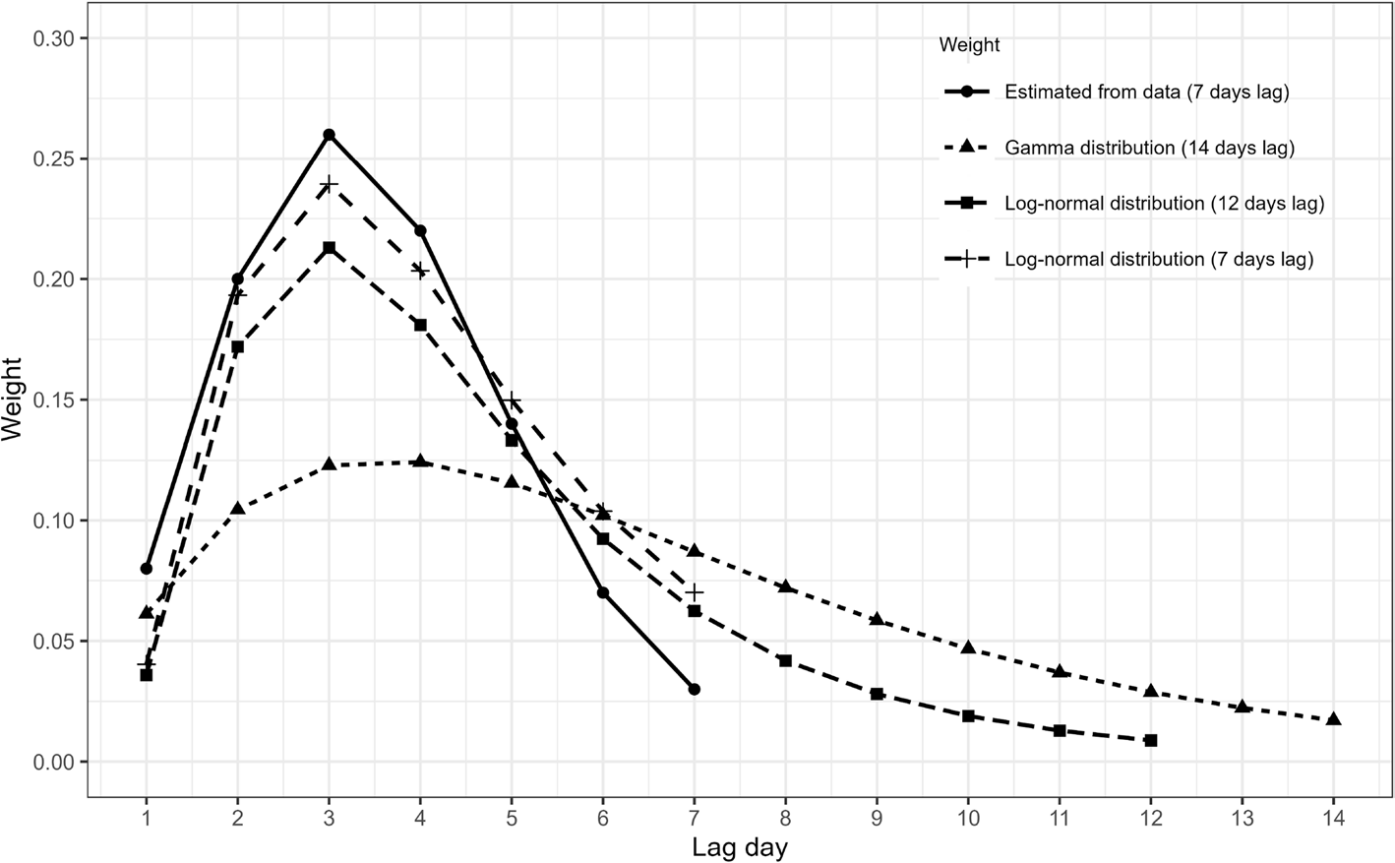
Lag distribution estimated from the data, a gamma distribution and two log-normal distributions

The comparison between the estimated lag distribution from our model and from literature is illustrated in Fig. 5. This lag distribution reflects the distribution of the serial interval (i.e., the time between symptom onset days of two paired cases). Parameter estimates from using an alternative definition of the mobility matrix, the adjusted Stringency Index, and from fixing the serial intervals are given in Tables A1 and A2 in the Appendix, respectively. Similar conclusions are found in these analyses.

**Fig. 6:**
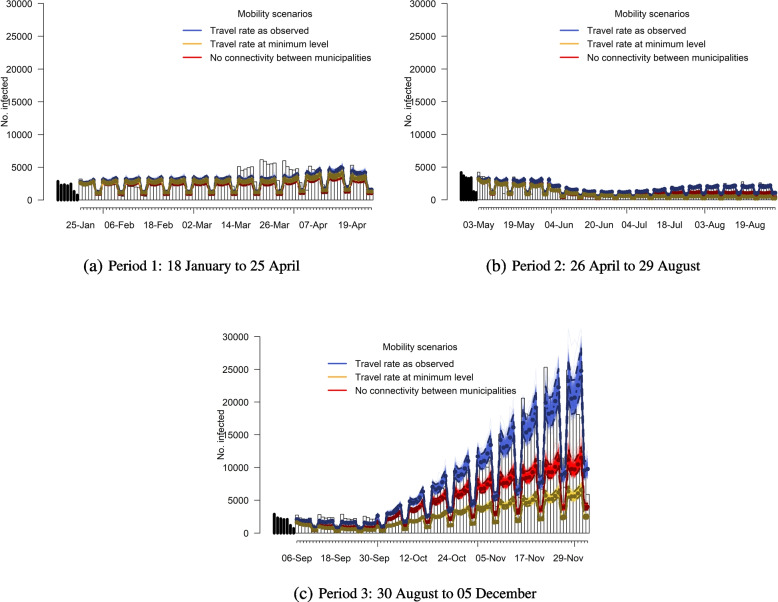
Comparison of simulation-based predictions for the number of infections in three mobility scenarios: travel rate as observed (blue), travel rate at the minimum level observed (yellow), and no connectivity between municipalities (red) against the observed values (bar chart). In each simulation, the lower and upper lines represent the pointwise 2.5% and 97.5% simulation-based percentiles for each day; the middle line displays the mean values

Figure 6 illustrates the impact of travel by means of three simulated scenarios for three periods: 18 January to 25 April (period 1), 26 April to 29 August (period 2) and 30 August to 5 December (period 3). In the first scenario (travel rate as observed), the simulation was able to capture the observed epidemic curve despite a slight underestimation in period 1 and overestimation in period 2 as compared to the observed cases (93.9% and 114%, respectively). In the second scenario (reduced international travel) and third scenario (reduced national travel), the simulation clearly shows that restricting international travel and local mobility would greatly reduce the epidemic growth. No connectivity among municipalities would imply the largest effect on the spread in period 1, whereas in period 2 and 3, minimizing incoming international travelers would be the most effective. Specifically, a travel rate fixed to the minimum level that was observed would have prevented 11.4% of cases in period 1 up to 68.3% in period 3. When there is no connectivity among municipalities, we also observe a decrease in cases with the largest reduction being 43.8% of cases in period 3 as compared to the first scenario (see Table A3).

## Discussion

Using the endemic-epidemic modelling framework, we performed a detailed analysis concerning the spatio-temporal characteristics of the COVID-19 pandemic at municipality level in Belgium. We successfully investigated the impact of different levels of human mobility, both between municipalities as well as by incoming travellers, on the daily incidence during 2021, while considering important covariates, such as vaccination on the one hand, and the Stringency Index as a proxy for the effect of NPIs that are in place.

From the model results, we conclude that a relatively high fraction of cases originated from local transmission within the municipality itself, suggesting that endogenous transmission contributed the most to the COVID-19 epidemic, at least during 2021. The spatio-temporal contribution was relatively low in the earlier part of the year. However, it increased during summer, the time when travel related policies became less stringent, suggesting that a larger fraction of cases during this period were imported from other areas. In certain municipalities, the progression of the disease remained local, though an elevated contribution of the neighbourhood component was visible in a part of the Brussels Capital Region, especially in the second half of the year (Appendix Fig. A3). This is plausible since intensive connectivity between municipalities on the one hand and a higher fraction of incoming travelers per capita were observed in the Brussels Capital Region.

It is suggested that differences in levels of travel played a role on daily confirmed cases. This hypothesis was confirmed by constructing three counterfactual scenarios, each with a different level of mobility. Reducing the mobility of individuals in the second and third counterfactual scenario corresponded with an attenuated COVID-19 case growth. Previous studies have investigated the relationship between disease data and human movement, but these studies were more focused on the areal mobility matrices, which illustrate the origin–destination connectivity between geographical units [1, 12, 40–42]. Our work is one of a few studies that account for mobility patterns outside of the study settings, as a potential source of infection [43, 44]. Nevertheless, regardless of the transmission model applied, the reduction on travel is effective in reducing the spread and size of epidemics. While using aggregated mobility flows can help estimating reductions in incidence at the population level, it would be intriguing to examine the patterns of spread influenced by changes in individual-level travel behaviors. While not without challenges in data acquisition, one might take into account the finer resolution of mobility data that involves information on individual contacts or clustering patterns. Adopting these mobility and travel behaviors may benefit to identify disease hotspots, adapt appropriate local-level regulations and control strategies.

From the estimated model coefficients, we found strong evidence of commuter-driven spread when we accounted for the unit-specific population in the neighbourhood component, indicating that regions with a larger population are expected to attract a bigger number of cases from their neighbors. The existence of such an agglomeration effect was also confirmed in previous studies [14, 15, 22, 45]. We identify that vaccination coverage of the first dose in Belgium did not have a significant association with fewer case counts. In endemic-epidemic models of areal count time series (so-called hhh4 models within the *surveillance* package in R), the rate of disease diffusion is allowed to be proportional to the number of susceptible people, which can be inferred from the number of non-vaccinated individuals [21]. In our study, only the local coverage of the first COVID-19 vaccine dose was used in the model. The uncertainty about the true immunity levels in the population inferred from one-dose coverage should be noted. By including the proportion of unvaccinated individuals in the model, we assumed that the susceptibility level in the population over time is proportionate to the time-varying proportion of unvaccinated individuals. More specifically, immunity in unvaccinated individuals as a result of previous infection with SARS-CoV-2 would imply an overestimation of the time-varying susceptibility at the population level when solely informed by vaccination coverage. However, when assuming that a constant fraction of unvaccinated individuals has immunity within the study period (and if waning of (vaccine-induced) immunity is limited), the fraction of unvaccinated individuals is proportional to population susceptibility. We acknowledge that this is a strong assumption and an investigation of the sensitivity of our results with regard to this assumption is considered a topic for future research. Besides, for a disease such as measles, the assumption that depletion of susceptibles due to vaccination decreases the transmission risk is a plausible one, but it arguably does not hold for COVID-19 given waning of humoral immunity with time since vaccination, the need for a second dose to maintain high protection against severe disease and the fact that vaccination does not completely prevent infection. Given age-specific differences in the fraction of the population that is protected against SARS-CoV-2 infection due to prior exposure or vaccination [46, 47], an age-stratified model would be a better approach to evaluate the impact of vaccination on the disease dynamics. To our surprise, we found that the positive association between stringency of NPIs and the new cases was not significant. Although recent studies indicated that this factor significantly contributed to the lower transmission of cases [14, 15], such a beneficial effect was not observed in this context. One possible reason is that the Stringency Index provides a surrogate picture of control measures by assigning one estimated value for the entire Belgian population. A more granular perspective would provide additional information about the role and impact of NPIs in controlling the epidemic. For example, it would be interesting to replace the Stringency Index with local-level indices to investigate the impact of NPIs. Furthermore, compliance to intervention measures and its variation across Belgium is not captured by this measure. Stringency should ideally be combined with information on adherence to NPIs and with social contact data. In this way, studying the impact of NPIs on space-time spread of the virus is a relevant topic of further research, conditional on the availability of sufficient data. Another aspect that may contribute to this controversial result is the fact that we used a lag of 7 days in the model. It might be unlikely that the impact of control measures in reducing transmission will be noticeable within a week, as it may take 9-12 days or longer to manifest in the trajectory of the epidemics [40]. Additional research could investigate the correlation between the delayed effects of NPIs and changes in disease incidence, for example, by examining the rate of change in reproductive number [48]. Furthermore, we discovered that by fitting a model with the Stringency Index, excluding travel restrictions, we found that dependencies between the Stringency Index versus the national mobility and the international travel rate in our model had minimal influence on model estimates. The results of this sensitivity analysis are shown in Table A2 in the Appendix.

During our study period, the Alpha variant was most prevalent over the largest part of the first half of 2021, while the Delta variant was dominant in the second half of the year. Although the Beta and Gamma variants were concomitantly identified over the first half of 2021, they did not play an important role in subsequent waves of SARS-CoV-2 variants observed in Belgium [30, 49]. Furthermore, previous studies have shown that both the Alpha and Delta variants have 50%-70% higher transmissibility compared to the pre-existing variants, and they impacted individuals with similar demographic and comorbidity characteristics [49, 50]. The emergence of variants of concern (VOCs) such as Alpha and Delta variants, which are capable of immune evasion in different molecular mechanisms, may suggest different transmission risks. Adeyinka et al., 2022 suggested that the evaluation of the spread of VOCs should account for other factors such as vaccine uptake, the strength of control measures that are in place, and mobility patterns [51]. However, due to the small size of Belgium as a country, significant geographical variation in the prevalence of VOCs across different provinces is unlikely, as least during our study period - even though there may be some regional differences. Moreover, our model considered different time periods that did not precisely align with the periods when certain VOCs became more dominant. Although it was feasible to adjust the time periods to match, this did not lead to improvement in the model, but rather provoked convergency issue. Therefore, the findings of this study should be interpreted while bearing in mind these VOCs.

A notable strength of our modelling strategy is that we were able to mine the mobile network activity to establish the spatial dependence of COVID-19 transmission. Mobility data has gradually shown its potential and importance in (spatial) analyses of infectious diseases [1, 24, 40, 43]. Our study has underscored its usefulness in modelling the connectivity between municipalities. In other words, they allow the modeller to construct between-municipality weights. Although a power law, which describes the short-time human movement behavior when there is missing information about travel, improved model fit in terms of AIC (Appendix Table A2), it makes more sense to apply the mobile network data to the model as it reflects the “true” activity logs of individuals geographically. In addition, we used the daily case counts and used observations to 7 days back in time. This allowed us to avoid biases in serial interval assumptions when using the aggregated data (in weekly data, for example) [33]. The estimated lag distribution in our study was in line with findings in the studies of Grimée et al. (2022) and Ssentongo et al. (2021) [3, 14]. The sensitivity analyses with different literature-based serial intervals also showed moderate stability of our results. Furthermore, the covariates used in the model (e.g., travel rate, vaccine coverage, stringency index) were time-varying. This approach is particularly important in the context of COVID-19, where policies and interventions tend to be highly variable, potentially over short periods of time.

Several limitations need to be mentioned. First, our mobile network data was not collected during the whole study period. This may lessen the representative quality for the human activity logs in the time period under investigation. Besides, we used a static spatially-dependent network to capture the interaction between localities rather than the time-dependent connectivity matrix, which may better describe the commuting behavior of individuals. Second, we assumed a homogeneous mixing scenario, which prevents us from examining the interaction between individuals in disease spread. Endeavors have been made to clarify the transmission dynamics in subgroups (age, gender) using social contact data [52–54]. Nowadays, such disease-related data and data on human behavior are collected and stored more systematically with high resolution and subject to specific circumstances. Future extensions of the EE framework would ideally address some or all of these limitations.

## Conclusions

In conclusion, our research focus centered on capturing the COVID-19 diffusion at municipality level in Belgium through a space-time dynamic model and identifying covariates that serve as contributing factors to the virus spread. Even though transmission between municipalities was observed during the study period, local transmission was dominant. We highlighted the positive association between the mobility data and the infection spread over time. Insight from our study can assist in decisions of public health policies, particularly when the disease is likely influenced by human movement.

## Supplementary Information

**Additional file 1:** **Appendices.**

## Data Availability

The datasets generated and/or analysed during the current study are available from https://github.com/OxCGRT/covid-policy-tracker/tree/master/data (stringency index), https://epistat.sciensano.be/covid/ (vaccine coverage) and https://statbel.fgov.be/en (population). The datasets for case counts, national and international mobility are available from the authors upon reasonable request (contact Minh Hanh Nguyen via minhhanh.nguyen@uhasselt.be or Thi Huyen Trang Nguyen via thihuyentrang.nguyen@uhasselt.be) and with permission of Sciensano.

## Abbreviations

SARS-CoV-2: Severe Acute Respiratory Syndrome Coronavirus 2
COVID-19: Coronavirus Disease 2019
NPIs: Non-pharmaceutical interventions
OxCGRT: Oxford Coronavirus Government Tracker
EE: Endemic-epidemic
AIC: Akaike Information Criteria
PLF: Passenger Locator Form
