# Supplementary material for: The impact of national and international travel on spatio-temporal transmission of SARS-CoV-2 in Belgium in 2021
Source: BMC Infect Dis. 2023 Jun 24;23:428. doi: 10.1186/s12879-023-08368-9 (PMC10290350; doi:10.1186/s12879-023-08368-9)
Supplement: Supplementary file 1 — Additional file 1: Appendices. [file 12879_2023_8368_MOESM1_ESM.pdf]

Appendix

Table A1: Model parameter estimates with corresponding 95% confidence intervals (CIs) (fit with a mobility matrix defined by the sum of the initial mobility matrix and its transpose).

	Local epidemic			Global epidemic			Endemic		
	Estimate	95% CI		Estimate	95% CI		Estimate	95% CI	
Intercept	0.53	0.47	0.59	2.19	0.78	6.13	4.95	3.88	6.30
Weekend	0.34	0.33	0.35	0.17	0.12	0.23	0.52	0.50	0.55
Population	1.03	1.02	1.04	0.78	0.72	0.84	-	-	-
Susceptible proportion	1.00	0.98	1.03	-	-	-	1.24	1.17	1.31
Travel rate	1.07	1.05	1.09	1.17	0.99	1.39	1.72	1.65	1.78
Travel rate^2	0.97	0.96	0.98	0.39	0.31	0.49	0.97	0.96	0.98
Stringency Index	1.01	1.00	1.01	0.98	0.97	0.99	1.01	1.01	1.02
Months Jun-Sep	0.86	0.83	0.89	1.14	0.93	1.40	0.34	0.31	0.37
Months Oct-Dec	1.41	1.35	1.46	0.77	0.60	0.99	0.68	0.61	0.75
AIC	755057.2								

Table A2: Parameter estimates from sensitivity analyses,using Passenger Locator Form data,fixed serial intervals with gamma(2.29, 0.36) and lognormal(1.38,0.563) distribution, a power law matrix for connectivity, and the adjusted Stringency Index

		PLF data			Ud (gama dist.)			Ud (lognorm 12 days)			Ud (lognorm 7 days)			Power law weights			Adjusted stringency index		
Local epidemic	Intercept	0.65	0.57	0.74	1.07	0.96	1.19	0.91	0.80	1.02	0.81	0.71	0.91	0.36	0.31	0.41	1.33	1.18	1.51
	Weekend	0.33	0.32	0.34	0.35	0.34	0.35	0.32	0.32	0.33	0.32	0.32	0.33	0.37	0.36	0.38	0.31	0.31	0.32
	Population	1.00	0.99	1.01	0.99	0.98	0.99	0.99	0.98	1.00	0.99	0.98	1.00	1.09	1.08	1.10	0.99	0.98	1.00
	Susceptible prop.	0.98	0.96	1.00	1.00	0.98	1.03	1.00	0.98	1.03	1.00	0.98	1.03	1.04	1.01	1.07	1.02	0.99	1.04
	Travel rate	0.73	0.66	0.81	1.06	1.04	1.08	1.03	1.01	1.06	1.03	1.01	1.05	1.14	1.12	1.16	1.01	0.99	1.03
	Travel rate^2	1.33	1.17	1.51	0.97	0.97	0.98	0.97	0.96	0.98	0.97	0.96	0.98	0.96	0.96	0.97	0.98	0.97	0.99
	Stringency Index	1.00	1.00	1.01	1.00	1.00	1.00	1.00	1.00	1.00	1.00	1.00	1.00	1.00	1.00	1.00	1.00	0.99	1.00
	Months Jun-Sep	0.95	0.91	0.98	0.79	0.76	0.81	0.84	0.81	0.87	0.86	0.82	0.89	0.79	0.76	0.82	0.81	0.78	0.84
	Months Oct-Dec	1.51	1.45	1.58	1.27	1.23	1.32	1.37	1.31	1.42	1.43	1.37	1.49	1.13	1.08	1.18	1.23	1.17	1.30
Global epidemic	Intercept	0.05	0.03	0.09	0.01	0.00	0.02	0.02	0.01	0.04	0.03	0.02	0.07	0.00	0.00	0.00	0.04	0.02	0.07
	Weekend	0.79	0.73	0.85	1.12	1.00	1.24	0.96	0.87	1.05	0.86	0.79	0.93	0.19	0.17	0.21	0.86	0.79	0.94
	Population	1.14	1.10	1.18	1.17	1.11	1.23	1.15	1.10	1.20	1.14	1.09	1.18	1.95	1.90	2.01	1.14	1.09	1.18
	Travel rate	2.89	2.12	3.92	1.52	1.40	1.66	1.33	1.24	1.42	1.22	1.15	1.31	0.88	0.84	0.93	1.22	1.15	1.30
	Travel rate^2	0.59	0.42	0.84	0.98	0.97	1.00	1.00	0.98	1.01	1.01	0.99	1.02	0.93	0.90	0.96	1.00	0.99	1.02
	Stringency Index	1.00	1.00	1.01	1.02	1.01	1.03	1.02	1.01	1.03	1.01	1.00	1.02	0.99	0.99	0.99	1.01	1.00	1.02
	Months Jun-Sep	2.43	2.13	2.77	1.84	1.42	2.39	1.92	1.59	2.32	1.94	1.64	2.29	0.73	0.67	0.79	1.86	1.60	2.17
	Months Oct-Dec	1.08	0.90	1.30	1.07	0.80	1.42	1.11	0.89	1.37	1.08	0.89	1.31	1.04	0.96	1.13	1.22	0.97	1.53
Endemic	Intercept	2.60	2.07	3.28	1.71	1.26	2.32	3.27	2.50	4.27	3.99	3.10	5.15	0.16	0.08	0.32	2.91	2.29	3.69
	Weekend	0.32	0.30	0.34	0.43	0.40	0.47	0.38	0.36	0.41	0.38	0.36	0.40	0.98	0.91	1.06	0.36	0.34	0.39
	Susceptible prop.	0.71	0.68	0.75	1.10	1.01	1.19	1.07	0.99	1.15	1.05	0.98	1.13	1.51	1.38	1.65	1.05	0.98	1.12
	Travel rate	2.13	1.62	2.79	1.80	1.70	1.90	1.60	1.52	1.69	1.55	1.48	1.63	2.16	2.04	2.29	1.55	1.47	1.62
	Travel rate^2	0.56	0.36	0.87	0.85	0.83	0.88	0.91	0.89	0.94	0.93	0.91	0.95	0.95	0.93	0.96	0.94	0.92	0.96
	Stringency Index	1.02	1.01	1.02	1.03	1.02	1.03	1.02	1.02	1.02	1.02	1.01	1.02	1.05	1.04	1.06	1.02	1.02	1.03
	Months Jun-Sep	0.29	0.27	0.32	0.40	0.36	0.44	0.32	0.30	0.35	0.30	0.28	0.33	1.09	0.88	1.35	0.32	0.29	0.34
	Months OCt-Dec	0.89	0.80	0.99	1.17	1.02	1.34	0.93	0.83	1.05	0.85	0.76	0.95	1.80	1.39	2.32	1.10	0.96	1.25
AIC		754913.7			753242.1			754079.5			754807.2			752615.3			754412.3		

Table A3: Predicted cumulative cases, and the 2.5% and 97.5% quantiles from three counterfactual scenarios. The second and third scenarios were compared to the first scenario and presented in percentages

Scenario	Period 1 (18 January - 25 April)			Period 2 (26 April - 29 August)			Period 3 (30 August - 05 December)		
	Predicted (%)	2.5%	97.5%	Predicted (%)	2.5%	97.5%	Predicted (%)	2.5%	97.5%
Travel rate as observed	256,086 (100%)	248,057	264,522	199,751 (100%)	192,578	207,548	696,490 (100%)	651,185	747,927
Travel rate at minimum level	226,966 (88.6%)	220,275	233,748	108,380 (54.3%)	104,868	111,886	220,615 (31.7%)	207,238	235,268
No connectivity between municipalities	203,044 (79.3%)	198,147	208,490	131,701 (65.9%)	128,545	134,807	391,458 (56.2%)	371,270	415,686

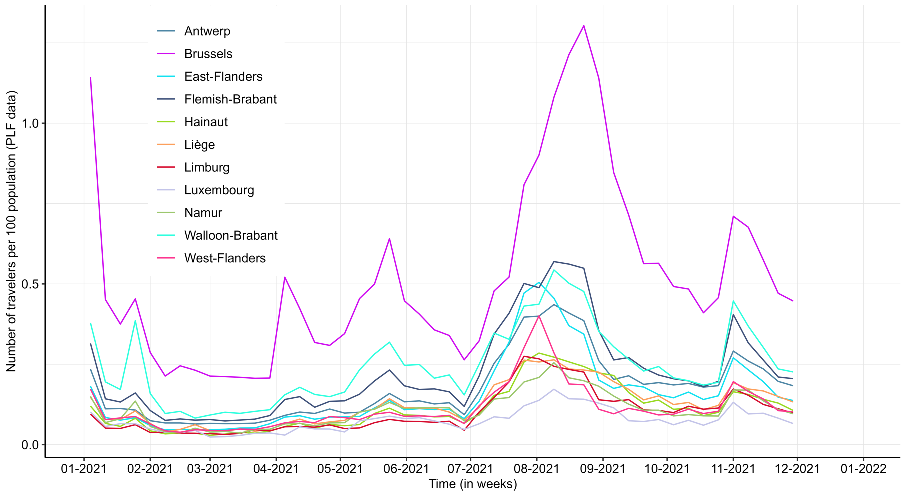

Figure A1: The number of incoming travelers per 100 population by province derived from Passenger Locator Form data

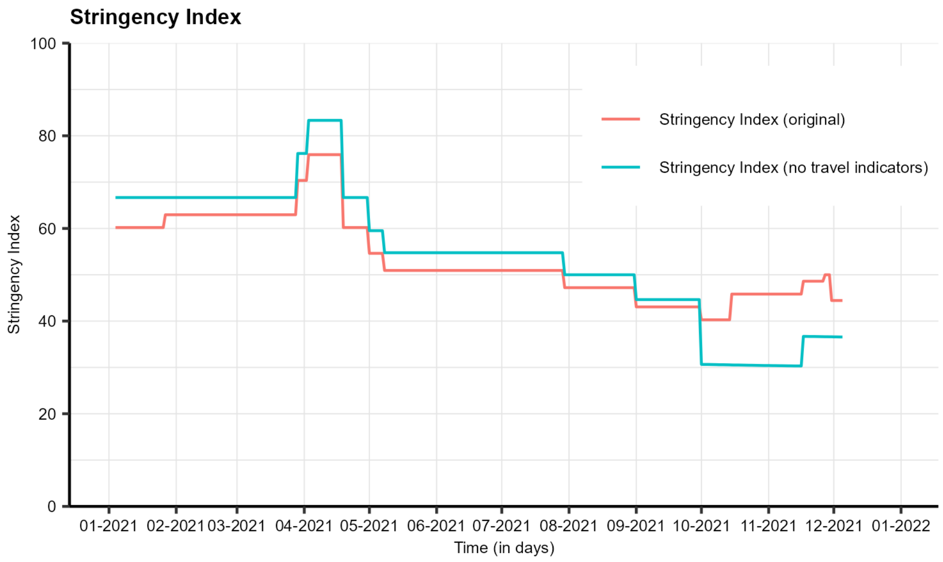

Figure A2: Comparison of two Stringency Indices, with and without travel indicators

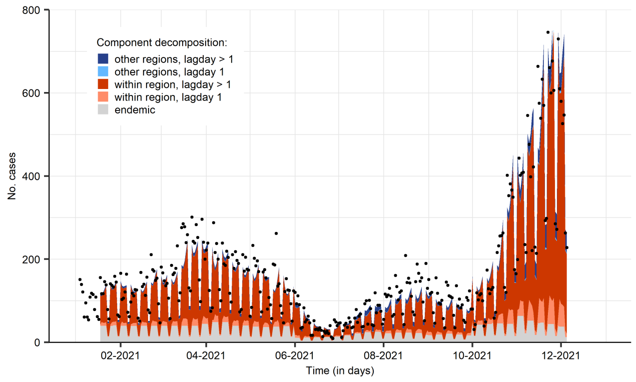

(a) Municipality 11002 in Antwerp (52 338 reported cases)

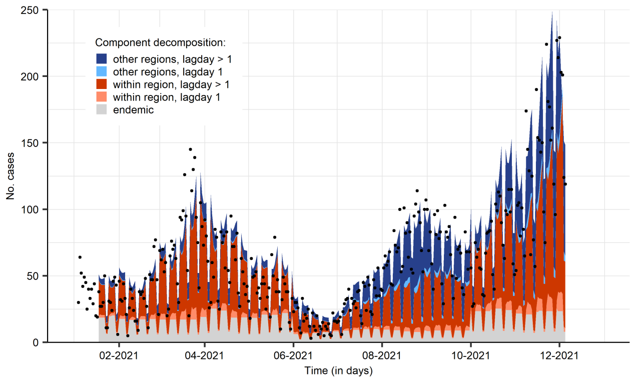

(b) Municipality 21004 in Brussels (20 121 reported cases)

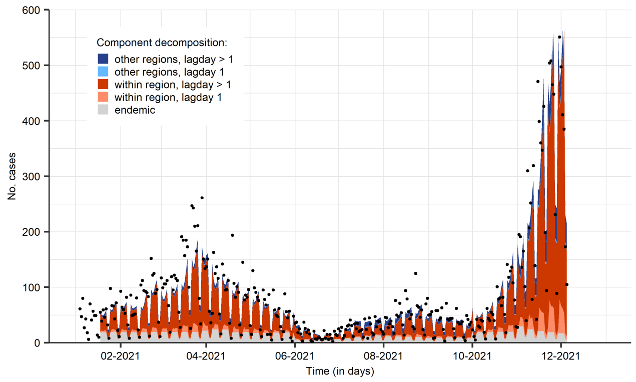

(c) Municipality 44021 in East-Flanders (27 429 reported cases)

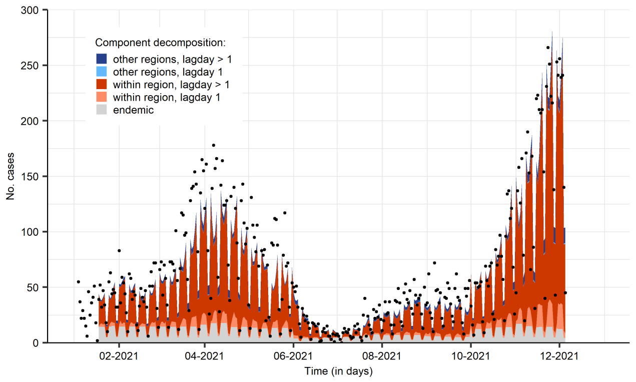

(d) Municipality 52011 in Hainaut (20 701 reported cases)

Figure A3: Fitted components of the model in selected municipalities where recorded the highest number of cases in Belgium. The dots indicate the observed number of daily infections

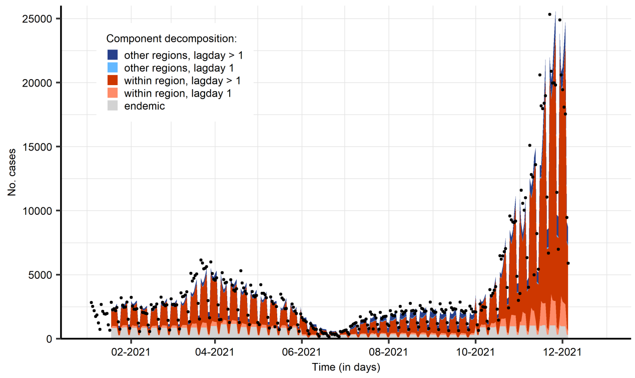

(a) Passenger Locator Form data

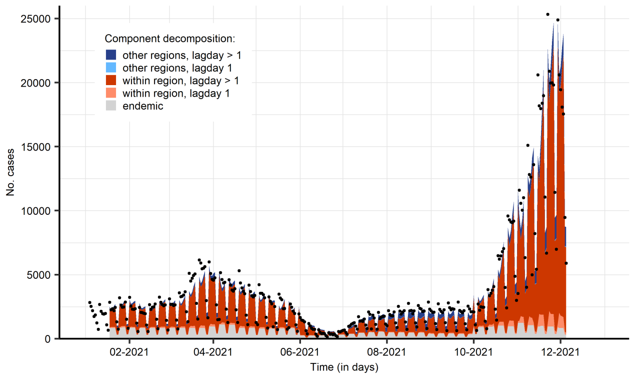

(b) Log-normal distribution of serial interval (7 days)

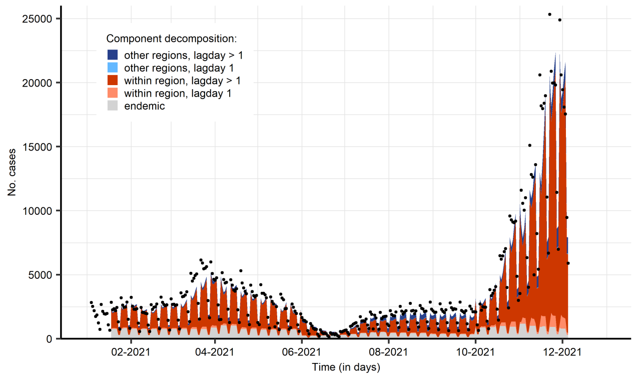

(c) Log-normal distribution of serial interval (12 days)

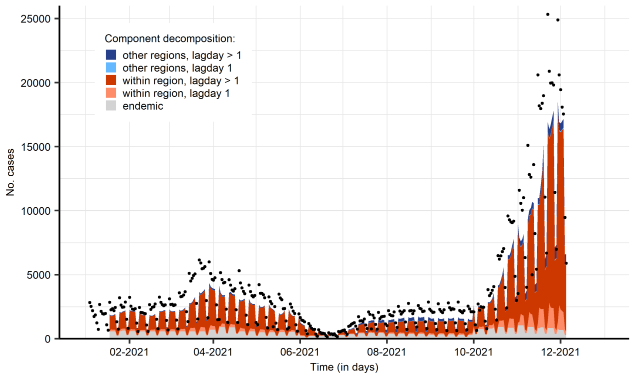

(d) Gamma distribution of serial interval (14 days)

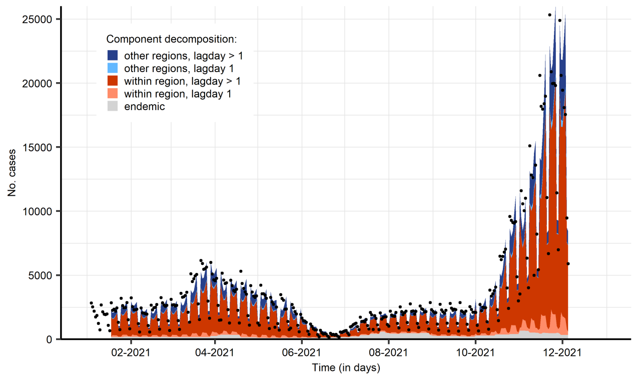

(e) Power law model

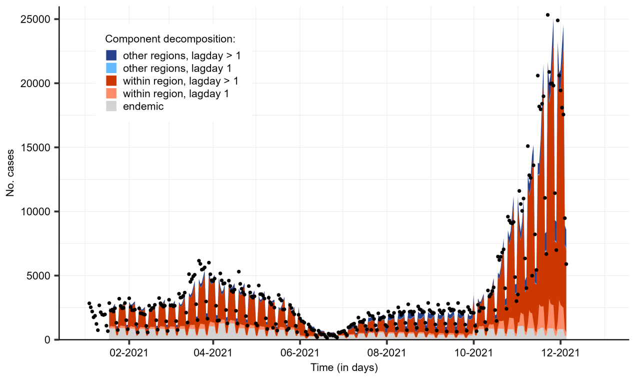

(f) Adjusted Stringency Index

Figure A4: Fitted components of the models in sensitivity analyses. The dots indicate the observed number of daily infections.

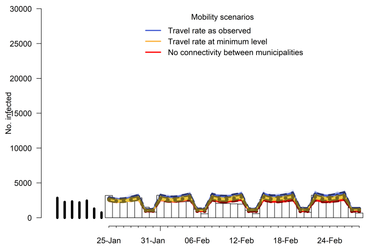

(a) February, 2021

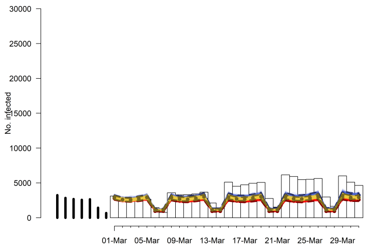

(b) March, 2021

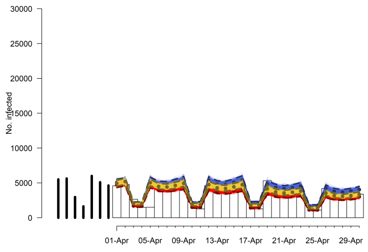

(c) April, 2021

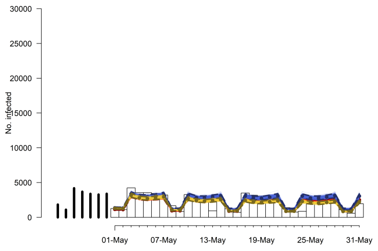

(d) May, 2021

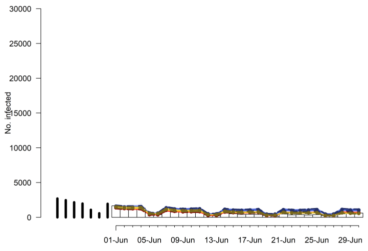

(e) June, 2021

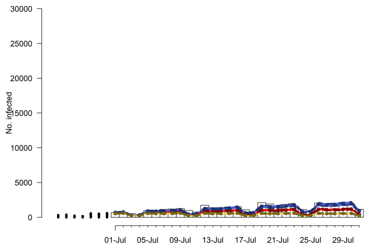

(f) July, 2021

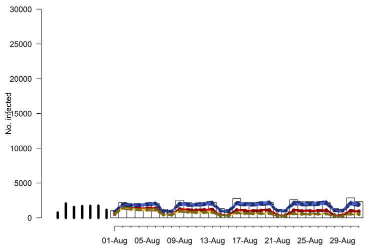

(g) August, 2021

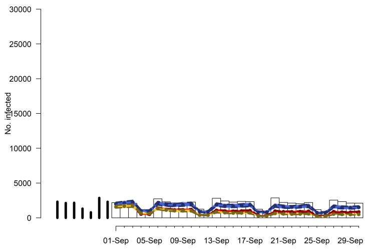

(h) September, 2021

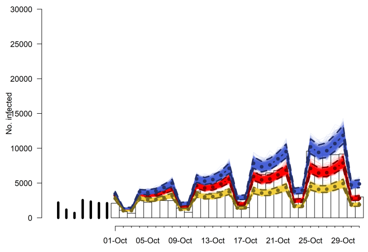

(i) October, 2021

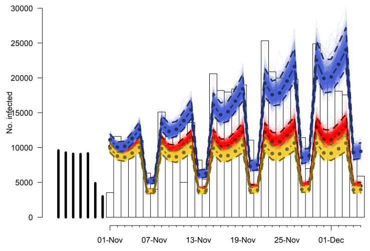

(j) November-December, 2021

Figure A5: Comparison of simulation-based predictions for the number of infections in three mobility scenarios in each month: travel rate as observed (blue), travel rate at the minimum level observed (yellow), and no connectivity between municipalities (red) against the observed values (bar chart). In each simulation, the lower and upper lines represent the pointwise 2.5% and 97.5% simulation-based percentiles for each day; the middle line displays the mean values.

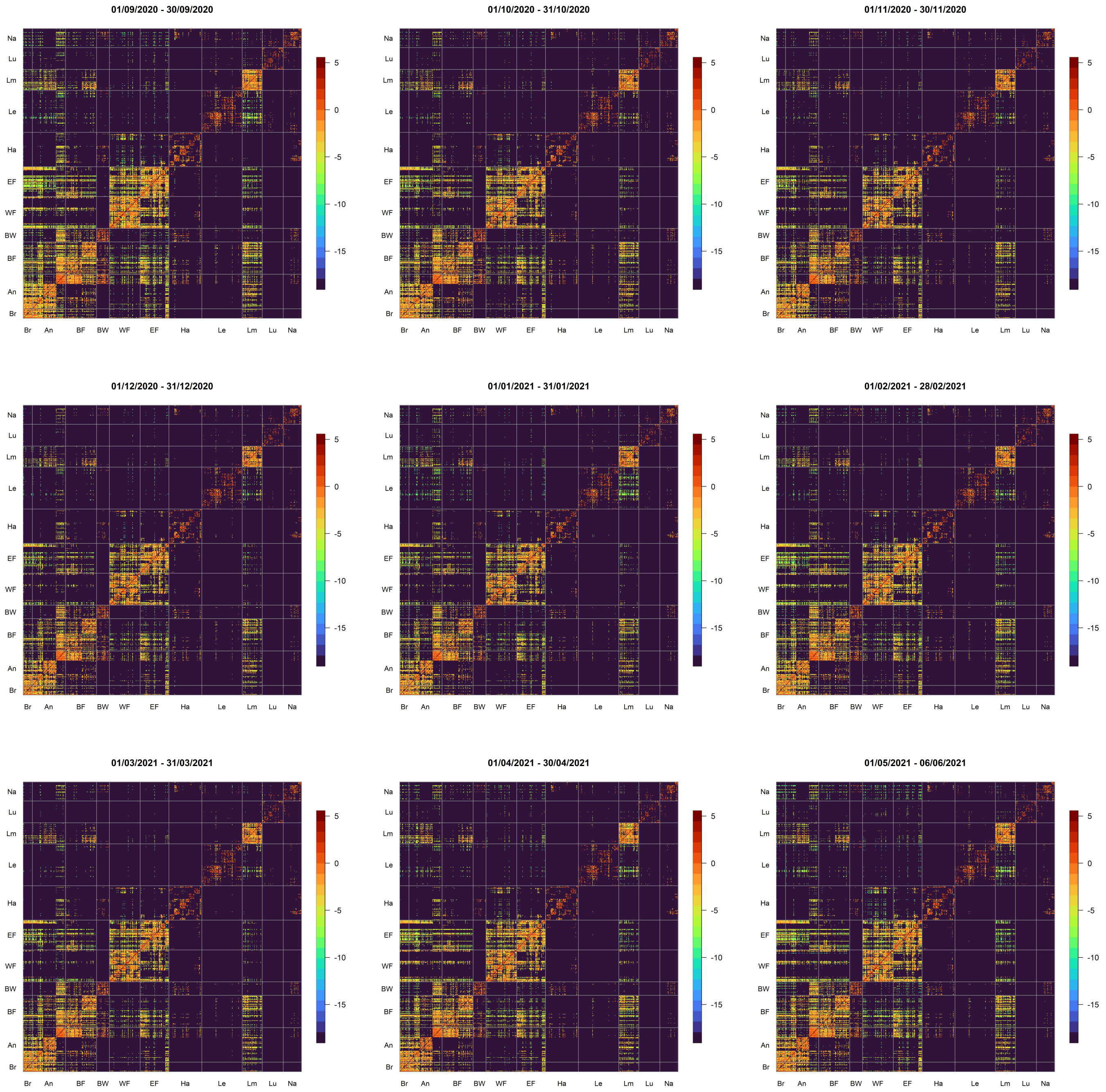

Figure A6: National mobility between September 1, 2020 and June 6, 2021 on a monthly basis. The y-axis represents the origin while the x-axis is the destination. Different municipalities are grouped according to the 10 Belgian provinces and Brussels-Capital Region (Brussels, Antwerp, Flemish Brabant, Walloon Brabant, West Flanders, East Flanders, Hainaut, Liège, Limburg, Luxembourg, and Namur).
